# Systematic Review and Meta-Analysis of Internal Jugular Vein Variants and Their Relationship to Clinical Implications in the Head and Neck

**DOI:** 10.3390/diagnostics14232765

**Published:** 2024-12-09

**Authors:** Pablo Nova-Baeza, Juan José Valenzuela-Fuenzalida, Rocio Valdivia-Arroyo, Emelyn Sofia Becerra-Rodríguez, Catalina Escalona-Manzo, Yesica Tatiana Castaño-Gallego, Ricardo Miguel Luque-Bernal, Gustavo Oyanedel-Amaro, Alejandra Suazo-Santibáñez, Mathias Orellana-Donoso, Alejandro Bruna-Mejias, Juan Sanchis-Gimeno, Héctor Gutiérrez-Espinoza

**Affiliations:** 1 Department of Morphology, Faculty of Medicine, Universidad Andres Bello, Santiago 7501015, Chile; pablo.nova@usach.cl (P.N.-B.); juan.kine.2015@gmail.com (J.J.V.-F.); rovaldiviaa04@gmail.com (R.V.-A.); e.catalina.n21@gmail.com (C.E.-M.); alejandro.bruna@unab.cl (A.B.-M.); 2 Departamento de Ciencias Química y Biológicas, Facultad de Ciencias de la Salud, Universidad Bernardo O’Higgins, Santiago 8370854, Chile; 3 Escuela de Medicina, Unidad Central del Valle del Cauca, Tuluá 763022, Colombia; emelyn.becerra01@uceva.edu.co (E.S.B.-R.); yesica.castano01@uceva.edu.co (Y.T.C.-G.); 4 Escuela de Medicina, Ciencias de la Salud, Universidad del Rosario, Bogota 111321, Colombia; ricardo.luque@urosario.edu.co; 5 Facultad de Ciencias de la Salud, Universidad Autónoma de Chile, Santiago 8910060, Chile; g.oyanedelamaro@gmail.com; 6 Faculty of Health Sciences, Universidad de las Americas, Santiago 8370040, Chile; alej.suazo@gmail.com; 7 Escuela de Medicina, Universidad Finis Terrae, Santiago 7501015, Chile; mathor9@gmail.com; 8 Department of Morphological Sciences, Faculty of Medicine and Science, Universidad San Sebastián, Santiago 8420524, Chile; 9 GIAVAL Research Group, Department of Anatomy and Human Embryology, Faculty of Medicine, University of Valencia, 46001 Valencia, Spain; juan.sanchis@uv.es; 10 One Health Research Group, Universidad de las Americas, Quito 170124, Ecuador

**Keywords:** jugular internal vein variations, aberrant jugular internal vein, encephalic venous drainage, clinical anatomy, anatomical variations

## Abstract

**Background**: The internal jugular vein (IJV) is a vascular structure that is responsible for the venous drainage of both the head and neck and is commonly found posterior to the internal carotid artery and adjacent to cervical lymph nodes or nerve structures such as the glossopharyngeal and accessory nerves. As a vagal nerve, it is an important reference point for surgical access in neck interventions and dissections. **Methods**: The databases Medline, Scopus, Web of Science, Google Scholar, CINAHL, and LILACS were searched until August 2024. Methodological quality was evaluated with an assurance tool for anatomical studies (AQUA). Pooled prevalence was estimated using a random effects model. **Results**: A total of 10 studies met the established selection criteria in this meta-analysis study. The prevalence of variants of the IJV was 3.36% (CI: 2.81–6.96%), with a heterogeneity of 94.46%. Regarding the subgroup analysis, no study presents statistically significant differences in the studies analyzed for this review. **Conclusions**: Knowing the IJV variants in detail will make early diagnosis useful, especially in surgeries in the neck region and in classic surgeries such as thyroidectomies and tracheostomies, among others. It will be important to know the position of the IJV.

## 1. Introduction

The internal jugular vein (IJV) is a vascular structure that is responsible for the venous drainage of both the head and neck and is commonly found posterior to the internal carotid artery and adjacent to cervical lymph nodes or nerve structures such as the glossopharyngeal and accessory nerves. As a vagal nerve, it is an important reference point for surgical access in neck interventions and dissections [1]. For this reason, the presence of anatomical variants in the IJV can make this access difficult, whether during the removal of a cervical lymph or lymph nodes in oncological surgeries, the installation of a central venous catheter, or interventional radiological procedures, among others [2]. The IJV can present various anatomical variants such as through fenestration or duplication; these variants can also modify the routes of structures close to the IJV [3]

The recognition of these anomalies should be decisive. Clinicians must be able to identify variations in IJV anatomy on diagnostic images to avoid incorrect interpretations that could lead to misdiagnosis. Additionally, during interventional procedures, such as the installation of central venous catheters, knowledge and expertise about aberrant venous anatomy can prevent complications such as venous perforations and thrombosis [1,2,3]. Intensive care professionals should also be alert to these abnormalities, because critically ill patients often require a central venous procedure for medication administration, parenteral nutrition, or hemodynamic monitoring, where such an IJV procedure is safe and effective. Given that the presence of an undetected anomaly could increase the risk of potential complications of death, in most cases, these variants are found during the surgical procedure itself and not before [3].

The objective of this meta-analysis was to determine IJV variants and how their presence could cause complications in the cervical and cranial regions.

## 2. Methods

### 2.1. PRISMA and PROSPERO Protocol

In order to carry out this study, we followed the guidelines of the PRISMA declaration for systematic reviews, complying with each of its points exhaustively [4]. To validate the originality of our review, we registered our review in the International Prospective Register of Systematic Reviews (PROSPERO) with the following validation code: CRD42024552875.

### 2.2. Eligibility Criteria

To select studies that met our research question and objectives, we used the following criteria: (a) study population: cadaveric dissection samples and live images of internal jugular vein variants; (b) results: prevalence report of internal jugular vein variations and their correlation with pathologies of the head and neck region, in addition to the anatomical variants that were classified or described based on normal anatomy and descriptions proposed in the literature; and (c) types of studies: retrospective or prospective observational research articles and case reports that involved only in vivo or cadaveric human samples. Letters to the editor were excluded from consideration.

### 2.3. Electronic Search

A literature search was conducted on the following databases: MEDLINE (via PubMed), Web of Science, Google Scholar, Cumulative Index to Nursing and Allied Health Literature (CINAHL), Scopus, and the Latin American and Caribbean Literature on Health Sciences (LILACS) from its inception to September 2024. The research strategy involved a combination of the following search terms: “variations jugular internal poisoning”, “Aberrant jugular internal”, “Encephalic venous drain”, “clinical anatomy”, and “anatomical varying” (without mesh), using the Boolean connectors AND, OR, and NOT. The search strategies for each database are available in Supplementary Table S1 (Table S1).

### 2.4. Study Selection

Two of the authors of this systematic review (RV-A and JJV-F) examined the studies by abstract or full title. The full text was obtained for the studies that each author considered potentially relevant for inclusion. If there was no consensus, a third reviewer (EB-R) was involved. To report a validity between the authors who evaluated the literature, we have applied the Kappa index which looks at the concordance between the results of included studies and which reported a mean value of 0.88, which indicates that there was a high concordance.

### 2.5. Data Collection Process

Two authors (JJV and CE) independently extracted data on the outcomes of each study. The following data were extracted from the original reports: (i) Authors and year of publication, (ii) geographical region, (iii) Age and sex (iv) prevalence (v) type of variants, (vi) clinical history vi) symptoms (vii) variants characteristics (viii) clinical implications.

### 2.6. Assessment of the Methodological Quality

To assess the risk of bias of articles that meet the eligibility criteria established by the authors, the methodological quality assurance tool for anatomical studies (AQUA) described and published by the International Evidence-Based Anatomy Working Group (IEBA) [5].

### 2.7. Statistical

The statistical data of this study were the calculation of the cumulative prevalence of the presence of internal jugular veins with some anatomical variant, for this calculation the JAMOVI software was used [6]. The degree of heterogeneity between the included studies was evaluated using the chi^2^ test and the heterogeneity statistic (I^2^). For the chi^2^ test, the *p* value proposed by the Cochrane collaboration was considered significant at 0.10. The I^2^ statistic values were interpreted with a 95% confidence interval [CI] as follows: 0–40% might not be important, 30–60% might indicate moderate heterogeneity, 50–90% might represent substantial heterogeneity and 75–100% might represent a significant amount of heterogeneity [7].

### 2.8. Subgroup Analysis

If an overestimation or underestimation of data from different subgroups is presented among the included studies, the most correct thing to do is to analyze the results by subgroups. Given the above, we have carried out a detailed subgroup analysis based on the information provided by the primary studies. Finally, the subgroups have been classified as follows: imaging samples, living patients and cadaver samples, geographic region, right or left side of the presence of the JIV variant, predominant sex of the variant and finally whether the variant is present uni or bilaterally, each subgroup will be analyzed individually and it will be seen if it presents statistically significant differences with the difference of means test, being statistically significant values less than *p* = 0.05.

## 3. Results

### 3.1. Included Articles

**Figure 1:**
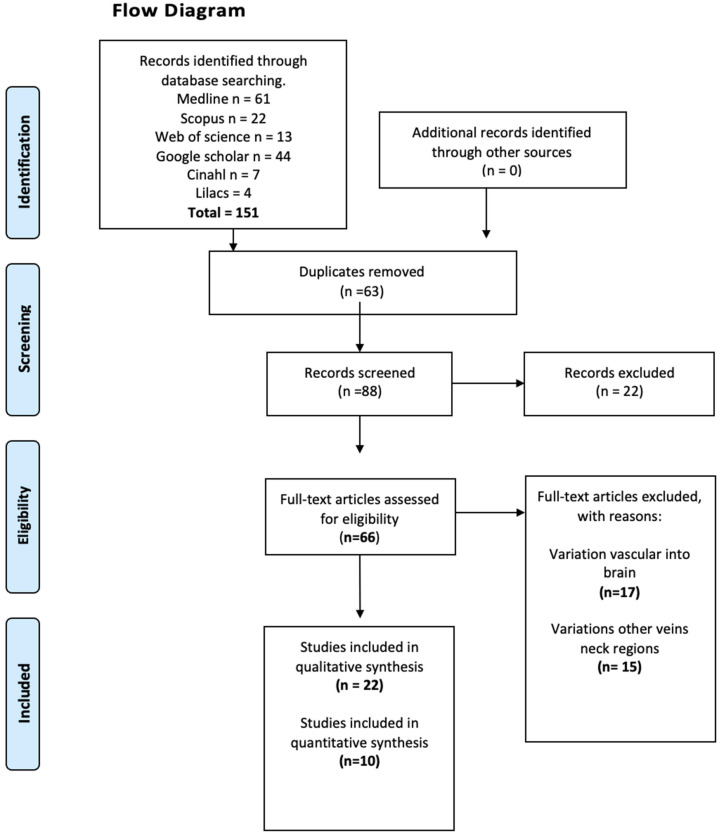
Search diagram for included studies.

The first result of this study gave a total number of studies that met our inclusion and exclusion criteria of 151 articles. Of these 151, when the full text was read in compliance with our criteria in Table 1, 63 studies were discarded, leaving a total of 88 studies. Of these, 88 studies were excluded (54 studies) due to discrepancies between the results reported or because they did not meet the data extraction criteria previously established by the authors. As a result, 32 articles [1,2,8,9,10,11,12,13,14,15,16,17,18,19,20,21,22,23,24,25,26,27,28,29,30,31,32,33,34,35,36,37] were included and considered for this study, where the data came from patients, imaging and cadavers (Figure 1).

### 3.2. Characteristics of the Studies and Population

Of the studies included in this review, 5 articles are from Asia, 1 from America, and 4 from Europe. Therefore, there are 3720 subjects, and of these 403 subjects presented a variant on the left side, corresponding to 3.97%, while 696 subjects presented a variant on the right side, corresponding to 4.45%. The average age of the subjects who reported the age was 53.2 years. Regarding sex in the included studies, we can say that in 6 studies they reported a female sex with a cumulative n of 432 subjects, while 5 studies showed that 672 subjects They were men, on the other hand, regarding the location of the anomaly, in 3 studies with 403 subjects, anomalies that started on the left side were reported and in 4 with 696 subjects, anomalies that started on the right side were reported.

### 3.3. Variants Description

The internal jugular vein originates at the base of the skull, in the jugular foramen, continuing with the sigmoid sinus. It descends through the neck into the carotid sheath, accompanied first by the internal carotid artery and then by the common carotid artery and cranial nerve X [45].

The internal jugular vein arises at the base of the skull, in the posterior part of the jugular foramen where the sigmoid sinus continues. The dilation that marks this origin is the superior bulb of the jugular vein, which occupies the jugular fossa of the temporal bone. It descends vertically, somewhat obliquely forward and laterally along the entire length of the neck. It ends behind the sternoclavicular joint, joining with the subclavian vein to form the brachiocephalic vein [46].

The jugular vein is a vein that is divided into internal and external, it is responsible for collecting non-oxygenated blood that comes from the head and is divided through the face, part of the neck until it reaches the heart, therefore it is formed the union of the deep veins of the head and neck.

Regarding anatomical relationships, it is located medially to the internal and common carotid arteries, in contact with cranial nerves IX, X, XI and XII. Previously throughout its course in the neck, it is covered by the sternocleidomastoid muscle. It receives blood from various tributary veins such as the facial vein, lingual vein, pharyngeal vein, superior thyroid vein, and in some cases, the middle thyroid vein. It also drains blood from the brain through the dural venous sinuses. The internal jugular vein ends posterior to the distal end of the clavicle where it joins the subclavian vein, forming the venous angle, where the thoracic duct (left side) and the right lymphatic trunk (right side) drain lymph into the venous circulation. which is collected from the entire body [47].

Fenestration of the internal jugular vein (IJV) can be partial or complete and occurs at different heights. The causes of these anomalies are varied and not yet completely understood, although there are several hypotheses that try to explain them as they are; The division of the IJV is generated by the posterior belly of the omohyoid muscle. Fenestration or duplication occurs due to inadequate condensation of the embryonic capillary plexus. Aberrant ossifications, caused by bone bridges, are responsible for venous partition, a topographic conflict between the Development of the IJV and the lateral branch of the accessory nerve suggests that the branching of the IJV is a consequence of growth occluded by the spinal accessory nerve (SAN) during development. Additionally, IJV can present as normal, dilated or ecstatic [45,46,47,48].

**Figure 2:**
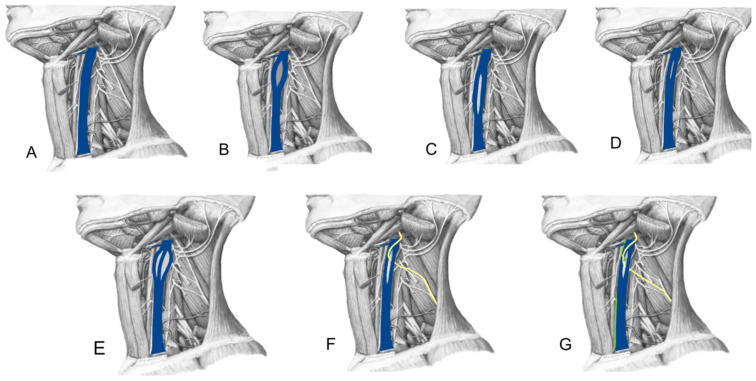
Variations JIV. Different anatomical variants in the internal jugular vein. (**A**) shows the internal jugular vein in its normal form in blue, while (**B**) presents a bifurcation in the jugular vein. In (**C**) a duplication of the jugular vein is shown internal unlike (**D**) which presents a fenestration, however, (**E**) exhibits a trifurcation of the IJV. On the other hand, (**F**) describes the variation of the blue vein in relation to the accessory nerve in yellow, which runs through the bifurcation of the internal jugular vein, a similar case occurs in (**G**) where the bifurcation of the internal jugular vein is traversed by both the accessory nerve in yellow and the vagus nerve depicted in green.

Agenesis of the internal jugular vein is an extremely rare condition that is usually asymptomatic and is discovered incidentally during imaging studies performed for other reasons. In some cases, a compensatory enlargement of the contralateral internal jugular vein, which must assume the function of venous drainage of the brain and neck, may occur, which may lead to its dilation. This enlargement can resemble a cervical mass, causing diagnostic confusion with pathologies such as tumors or lymphadenopathy in the neck. Hypoplasia of the internal jugular vein is a rare anatomical condition characterized by insufficient development of this vein, resulting in a reduced caliber. It can be unilateral or bilateral and affects venous drainage from the brain to the central venous system. Tortuosity of the internal jugular vein is a condition in which the vein has an abnormally meandering or coiled path, which alters venous flow. This alteration can cause venous stasis, increase venous pressure and the risk of thrombosis, and elevate intracranial pressure. Symptoms include recurrent headaches that vary in intensity depending on position, dizziness, vertigo, blurred vision, and papilledema due to intracranial hypertension. Tortuosity of the internal jugular vein can be congenital or acquired, often related to hypertension or old age. Communication between the internal jugular vein and the external jugular vein is a rare anatomical variation, usually detected by imaging studies such as Doppler ultrasound, magnetic resonance imaging (MRI) or computed tomography (CT). Phlebography can provide detailed visualization of this anomalous connection. This variation can cause venous congestion, leading to a feeling of fullness or pain in the neck and the formation of visible varicose veins. The presence of this anomalous communication can complicate procedures such as the insertion of central venous catheters, so it is essential that physicians are aware of this variation to avoid complications [45,46,47,48] (Figure 2).

### 3.4. Prevalence and Subgroups Analyzed

**Figure 3:**
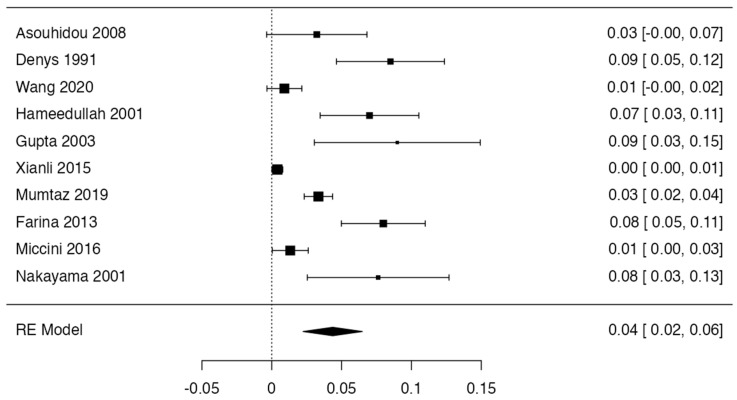
Forest plot of articles included in the prevalence meta-analysis [1,14,15,17,18,22,25,26,28,38].

**Figure 4:**
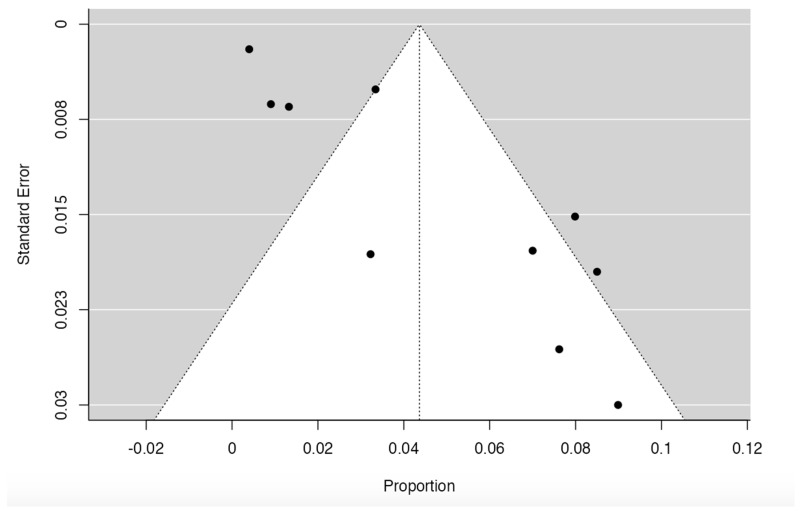
Funnel plot of articles included in the prevalence meta-analysis [1,14,15,17,18,22,25,26,28,38].

**Figure 5:**
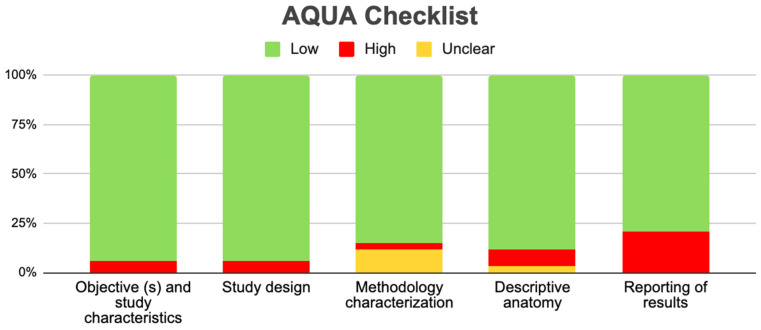
Risk of bias included studies [1,9,11,19,28,32,34].

Ten studies [1,14,15,17,18,22,25,26,28,38] were included (Table 2 and Figure 3) for the prevalence of internal jugular vein variants, presenting a prevalence of 3.36% (CI: 2.81–6.96%). The heterogeneity was 94.46%, which is high, and the sample was very heterogeneous between the groups analyzed (Figure 4). The funnel diagram was very heterogeneous for this sample due to the low number of studies included and the dispersion of data that these same studies presented in the included studies (Figure 5). In the subgroup analysis, we have grouped the studies with a prevalence not greater than 50%. The first subgroup analyzed in this study was cadavers versus images. For the cadaver subgroup, 3 studies were included [17,22,38], with a cumulative prevalence of 3.12% (CI: 1.87–8.16%) and a heterogeneity of 83.62%. In the imaging studies, 7 articles were included [1,14,15,18,25,26,28,38], which had a cumulative prevalence of 5.33% (CI: 2.12–7.31%) and a heterogeneity of 91.58%. For this subgroup analysis, there was no statistically significant difference between cadavers versus images with a *p* value = 0.165. Another subgroup analysis was for the continents from which the studies included in this review came. Five studies from Asia were included [1,17,22,28,38], which presented a cumulative prevalence of JIV variants of 4.44% (CI: 1.83–8.222%) and a heterogeneity of 94.42%. Four studies from Europe were included [15,25,26,44], presenting a cumulative prevalence of JIV variants of 4.88% (CI: 1.87–6.12%) and a heterogeneity of 88.73%. Only one study from South and North America was included [14], showing a cumulative prevalence of JIV variants of 8.5%. As it was only one study, no I2 or heterogeneity was presented for this sample. No study from the continents of Oceania and Africa was included. This subgroup did not show statistically significant differences in favor of any of the continents studied in the prevalence of IVJ variants, with a *p* value = 0.873. Regarding the right or left laterality of the IVJ variant. Firstly, for the presence of a left-sided IVJ variant, three studies were included [1,15,38] with a cumulative prevalence of 4.44% (CI: 1.11–8.92%) and a heterogeneity of 78.62%. For the right-sided IVJ variants, four studies were included [15,25,28,38], with a cumulative prevalence of 6.8% (CI: 2.11–9.45%) and a heterogeneity of 77.84%. For this subgroup, there were no statistically significant differences between the groups analyzed with a *p* value = 0.212. Regarding the sex subgroup of the included subjects, five studies [14,15,17,25,38] showed men with the JIV variant, which presented a cumulative prevalence of 2.52% (CI 1.82–4.11) while the heterogeneity of this comparison was 79.93%. Women with JIV variants were presented in six studies [1,14,15,17,24,38] with a cumulative prevalence of 1.48% (CI 0.65–2.51) and a heterogeneity of 91.22, for this subgroup analysis there was no statistically significant difference between men and women with the JIV variant *p*= 0321. Finally, the final subgroup analysis was whether the JIV variant was presented unilaterally or bilaterally, for this analysis three studies were included [14,17,22], for the unilateral form of the JIV variant studies were presented [17,22], with a cumulative prevalence of 1.65% (CI 0.99–2.35%) with a heterogeneity of 66.11%, finally only in one study [14]. The JIV variant was presented unilaterally, showing a prevalence of 1.12%, the result of the comparison of these groups showed that there is no statistical difference in the laterality of the internal jugular vein variant, with a *p* value = 0.455 (Table 3).

### 3.5. Risk of Bias of Included Articles

All studies included in this review were assessed using the AQUA tool (AaquaTools Inc, Rancho Cordova, CA, USA). Of the five domains offered by the AQUA table, only seven included studies presented a high risk of bias [1,9,11,19,28,32,34], which is when two or more domains present high bias, so these studies’ data should be interpreted with caution (Figure 5).

### 3.6. Clinical Considerations

Due to its size and accessibility, the internal jugular vein is commonly used for central venous access in medical procedures, in which it is preferable to use the right IJV, as it is generally larger and less tortuous. Conditions that can affect the internal jugular vein include thrombosis, phlebitis and external compression, all of which can have significant implications for neurological and vascular health [49]. The internal jugular vein (IJV) is a crucial anatomical landmark that is frequently located during various medical and surgical interventions. Its accurate identification is especially relevant in cervical lymph node dissection during oncologic surgery, in the insertion of central venous catheters, and in various interventional radiological procedures. The anatomical course of IJV can present significant variations; detailed knowledge of these possible variations is essential to minimize risks and improve them [50]. During embryonic development, neck veins, including the IJV, are formed through a complex remodeling process of primitive vessels. Irregularities in this process can lead to anatomical variations such as fenestration or duplication of the IJV. The phlebectomies seen in these cases could be the result of a weakness in the venous wall due to an underdeveloped or malformed muscle layer. This structural defect could make the vein more susceptible to dilation under pressure. [39]. Anatomical and embryological studies suggest that disruption in cellular and molecular signaling during vessel formation may contribute to these abnormalities. Genetic and environmental factors during pregnancy could also influence the correct formation of the venous wall and the differentiation of its layers. However, more research is needed to fully understand the precise mechanisms that lead to fenestration and duplication [1]. Better understanding these embryological bases not only has implications for clinical anatomy but may also help surgeons and radiologists better anticipate and manage these variations during medical procedures. This is crucial to avoid complications and ensure safer interventions [34]. In interventional radiological procedures, variability in the course of IJV can influence the choice of technique and the approach to the procedure. Therefore, familiarity with these anatomical variations not only improves patient safety, but also optimizes the effectiveness of interventions. Detailed understanding of the possible anatomical variations of the internal jugular vein is essential for healthcare professionals involved in oncological surgery, insertion of central venous catheters and interventional radiological procedures, as this contributes significantly to the risk reduction and success of the procedures. [12]. The presence of an unreported internal jugular vein (IJV) abnormality can have serious implications for radiological examinations and surgical procedures in the head and neck regions [1].

## 4. Discussion

In this systematic review and meta-analysis, we studied the variants of the internal jugular vein, focusing mainly on the drainage and course variants of the same. The main result we have found is that these variants are rare in the population, they present a considerable variability in their description and regions of appearance, which makes their study often controversial. In the presence of these variants, the lack of knowledge of them could cause complications in the diagnosis of different neck pathologies, including diagnostic complications of the thyroid gland, as well as intrasurgical complications of the different regions. Over time, they have also been associated with a greater predisposition to alterations in the venous drainage of tumors in the neck region, so knowledge of the variants and an early diagnosis of these same tumors in the neck region is necessary. For this last, more studies are also needed that can accept this relationship that a priori can infer some degree of association. In relation to the review of previous studies that were similar to this work, two meta-analyses associated with the internal jugular vein were found. The first review published by A G da Silva Correia (2023) [51], mentions the anatomical variants in relation to the accessory nerve and the internal jugular vein, which is a type of anatomical variant present in this study, in which four were identified and classified relationship patterns: type 1, the nerve is superficial to the vein; type 2, the nerve is deep to the vein; type 3, the nerve crosses the branches of the vein; Type 4, the nerve divides and its branches pass around the vein. And it also evokes the importance of recognizing this type of variant to avoid complications in neck dissections; however, this systematic review does not refer to direct variations of the internal jugular vein but rather of the accessory nerve. On the other hand, the systematic and meta-analytic review published by Yin (2020) [52] mentions the comparison of the anastomosis of the internal jugular vein system and the anastomosis of the external jugular vein system in free flaps for the reconstruction of the head and neck, by differentiating postoperative thrombosis or flap failures, obtaining that the IJV is the most efficient option, however there is no emphasis on any specific anatomical variant of the internal jugular vein, nor mention of the importance of the knowledge of these variants. Therefore, it is concluded that in no case are anatomical variations of the internal jugular vein identified, which corresponds to the purpose of this research, for this reason it is demonstrated that this study is completely original.

The chosen sample comes from 4 different continents, Europe, Asia, America and Oceania, with a total of 4482 subjects. The variation was studied in research based on cadavers, patients and images to get a broader view of the prevalence of the variant, as well as how and where to find it. The sample has an average age of 53.2 years, of the total subjects in the sample 22.5% are men, 16% are women and 61.4% do not report sex. Regarding the variant, based on the research we can see that the IJV presents diversity in terms of its location and anatomical relationships with the carotid artery and the spinal accessory nerve, among others; Variations in its length, diameter and fenestration or duplication in the neck were also studied, which leads to important clinical complications to be mentioned later. Regarding the quantitative results found in this review, we have found that the prevalence was associated with the description of an anatomical variant where it is defined that it must occur in a number no greater than 10% of the population, which is why it should be considered. As a rare variant in the neck region, with respect to the studies analyzed in prevalence, these presented a high heterogeneity, which should also make the results presented with caution. On the other hand, we have analyzed the studies that presented a quantifiable prevalence through subgroups, these subgroups being whether the sample studied was a cadaver or collection of images of patients, the next subgroup was by geographic region from where the sample was obtained where it was not showed a statistically significant difference towards some region where this variant will occur, for the relationship between the presence of the variant on the right side or the left side or its laterality (unilateral or bilateral), there were also no statistically significant differences. Finally, in the analysis of the sex of the sample, there were no statistically significant differences for the male or female sex. Therefore, these analyzes show us a priori that the presence of a variant of the internal jugular vein would not have any type of influence. race, sex or present early in patients since in the majority of them it will be asymptomatic. The internal jugular vein (IJV) is the largest vessel in the neck and head, it can present certain anomalies such as duplication or fenestration, this is a problem due to the extensive functions of the internal jugular vein. For head and neck surgeons, an anomalous IJV can represent a considerable challenge during surgery, as it is a key anatomical point in adjacent structures such as: the carotid artery, vagus nerve, SAN and cervical lymph nodes, this proximity with vital structures implies that any unexpected anatomical variation could increase the risk of inadvertent injury to these tissues. For example, during lymph node dissection or tumor resection, an abnormal IJV could be susceptible to damage, resulting in complications such as severe bleeding or damage to adjacent nerve structures [42,43].

In the field of radiology, recognition of these abnormalities is equally crucial. Radiologists must be able to identify variations in IJV anatomy on diagnostic images to avoid incorrect interpretations that could lead to erroneous diagnoses. Additionally, during interventional procedures such as placement of central venous catheters, awareness of aberrant venous anatomy may prevent complications such as venous perforations and thrombosis. Intensive care professionals should also be alert to these abnormalities. Critically ill patients often require central venous access. Cannulation of central venous accesses is a widely used procedure in medical practice, which is used for the administration of irritating or hyperosmolar drugs, the administration of fluids, the provision of parenteral nutrition, for hemodialysis and hemodynamic monitoring. However, these intravascular probes that are inserted into the large venous vessels of the thorax and abdomen should not be used for prolonged periods and the risk of arterial puncture is always evident. Therefore, safe and efficient access to the IJV is essential, and the presence of an undetected anomaly could complicate this process, increasing the risk of potential complications of death [1,41,53]. Understanding IJV abnormalities is important to avoid iatrogenic damage during neck surgery or central venous catheterization [12,40].

## 5. Limitations

This review was limited by the publication and authorship bias of the included studies. First, studies with different results that were in non-indexed literature in the selected databases may have been excluded. Second, there could be limitations in the sensitivity and specificity of the searches. Finally, the authors personally selected articles. All of this increases the probability of excluding potential cases from countries outside of Asia and North America that are not being reported in the scientific community.

## 6. Conclusions

The venous drainage of the neck is complex, the large vein that drains the neck and skull is the IJV, which can present important variations both in its drainage and its course, and finally in its relationships with neighboring structures such as the sheath. carotid artery, knowing this variant in detail is useful in the early diagnosis of neck pathologies, especially in surgeries in the craniocervical region. Above all, this could be accentuated in classic surgeries such as thyroidectomies, tracheostomies, among others in the anterolateral region. We believe that new primary studies will be able to better relate whether these variants can generate symptoms or other clinical considerations not widely addressed in patients with neck pathologies.

## Figures and Tables

**Table 1:** Characteristics of the included studies.

Author	N of Subjects and Diagnostic Methods	Variant	Prevalence	Age	Location	Male or Female	Clinical Considerations
Asouhidou et al., 2008 [38]	93 cadavers	Hypoplasia of the internal jugular vein	3/93	does not report	Greece	does not report	The location and diameter of the IJV can be associated with increased risk of vascular trauma during catheterization attempts, and may partially explain the lack of IJV cannulation in a number of patients
Benter et al., 2001 [8]	113 patients	anatomical variations in the internal jugular vein (IJV) and its posi- tion in relation to the common carotid artery (CCA) in cancer pa- tients.	36%	19 to 84 years.	Germany	Does not report	External landmark puncture may be difficult in a considerable number of patients since the IJV might not be situated in the presumed location anteriorly or laterally to the CCA. This study supports the use of ultrasound-guided techniques for central venous catheters particularly in hematological and oncological patients.
Buch et al., 2016 [9]	363 patients	Anatomical caliber of the course of the IJV	36.6% (Greater than 50% narrowing compared with a normalized average)	age range, 2–89 years; mean, 47.3 years.	EE. UU	191 female patients, 172 male patients	Chronic cerebrospinal vascular insufficiency is a proposed condition of intraluminal stenosis of the internal jugular vein (IJV) that impedes venous flow from the brain. Calculations of IJV stenosis are vague and described in veins with at least a 50% reduction in IJV caliber at a specific level.
Cho et al., 2020 [11]	1 patient	Left IJV on the medial side of the ACC.Left ACC crossed the Left IJV from medial to lateral at the level of the clavicle.	100%	74 years	does not reported	female	The left internal jugular vein has a higher possibility of anatomical variation than the right side. Therefore, the complication risk during cannulation is expected to be higher.
Contrera et al., 2016 [12]	3 Patients	Internal Jugular Vein Duplication and Fenestration	100%	51-year-old woman, 49-year-old man and 65-year-old man	EE. UU	1 female and 2 males	Internal jugular vein duplication and fenestration may risk iatrogenic injury to the vasculature and spinal accessory nerve during neck dissection.
Cvetko et al., 2015 [2]	1 cadaver	unilateral fenestration of the left IJV	100%	75-year-old	France	male	The IJV bifurcated into the medial and lateral branches at a level of 1 cm below the mandibular angle. Clinicians and surgeons performing neck vascular or reconstructive surgery should be made aware of this variation of the IJV in the hope of preventing inadvertent injury.
Denys et al., 1991 [14]	200 patients	underlying anatomical variations of the internal jugular vein	In five (2.5%) patients, the internal jugular vein was not visualized and was probably thrombosed. In six (3%) patients, the internal jugular The vein was unusually small.	between 7 and 52 years	USA	147 males and 53 females	These findings suggest that anatomical variation may partly explain the Inability to cannulate the internal jugular vein in certain patients
Lim et al., 2006 [21]	88 patients	Anatomical variations of the internal jugular veins and their relationship to the carotid arteries	100%	does not report	Australia	does not report	Anatomical variations of IJV in relation to the carotid arteries increase the risk of inadvertent carotid puncture.
Nayak et al., 2019 [29]	1 cadaver	variation of IJV is presented, Right IJV had an unusual vein joining it in the form of a ‘‘jug handle.’’ This vein arose from the junction of IJV and common facial vein (CFV), coursed down for 3 cm and joined IJV again.	100%	70 years approximately	India	male	Knowledge of this rare case could be useful to craniofacial surgeons, radiologists, and anesthesiologists. It might decrease the chances of iatrogenic bleeding during head and neck surgeries and radiologic procedures.
Prakash et al., 2006 [39]	10 subjects	The action of the omohyoid muscle on the hemodynamics of the internal jugular vein.	-	30 to 45 years.	France	5 males and 5 females	For some authors, the contraction of this muscle, by tightening the cervical fascia, favors jugular venous return. For others, contraction of this muscle compresses the jugular vein in its cervical course.
Jia et al., 2012 [19]	51 patients with CVST	Relationship between IJV anomaly and CVST, IJV anomaly and IJV reflux	61%) patients were with abnormal VJI. Types of IJV abnormality included ring stenosis in 19 cases (61%), hypoplasia in 9 cases (29%), thrombosis 2 cases (7%) and anomalous valve 1 case (3%).	does not report	China	does not report	Cerebral venous sinus thrombosis (CVST) is a unique disease. and frequently unrecognized stroke, with a estimates 5 cases per million annually, and represents 0.5–1.0% of all strokes.
Wang et al., 2020 [1].	221 patients	Fenestration and duplication of the IJV	0.9%. 2/221	40 y 43 years	China	2 females	JV fenestration refers to a bifurcation of the IJV that joins the subclavian vein proximally, while in IJV duplication both branches remain separate. In both cases, the spinal accessory nerve (SAN) crossed the window between the branches of the IJV.
Hameedullah et al., 2001 [18]	200 patients	The relationship of the right IJV with the carotid artery	n 49 cases the IJV was found in aberrant relation to carotid artery at the angle of the mandible (*p* value <0.05), 22 at the thyroid cartilage, 20 at the cricoid cartilage, and 46 at the supraclavicular area (*p* value < 0.05). In 93% of cases the IJV was found to be larger than the carotid artery.	37.33 ± 14.92	Pakistan	131 males and 69 females	Central venous cannulation is a commonly performed procedure by the anesthesiologists in the operating room and intensive care unit for various reasons like massive volume resuscitation, administration of vasopressor agents.
Lorchirachoonkull et al., 2012 [40]	100 adult patients presenting for elective cardiac surgery requiring venous catheterization	Anatomic Variations relevant to percutaneous central venous catheterization of the IJV	15%	>21 years	Singapore	84 males and 16 females	her difficulty was greater when catheterization of the left IJV was simulated than the right. The right IJV was significantly wider and had a larger AP diameter compared to the left IJV.
Wu 2023 [36]	56 patients	IJV variations		12 to 18 years	China	21 males and 35 females	IJV is a non-invasive, easy-to-determine predictor that accurately assesses fluid responsiveness in multiple clinical settings.
Horejsek 2023 [41]	54 patients	jugular internal vein (VYI)	50%	18 years	Czech Republic	43 males and 11 females	Promising predictive abilities of IJV culpability for spontaneously breathing patients and of IJV compliance for mechanically ventilated patients
Gupta et al., 2003 [17]	89 dissected adult cadavers	Variation in facial vein (FV) termination patterns in EJV	9%	does not report	India	81 males and 8 females	The common facial vein, which joins the IJV just above the carotid bifurcation, provides a useful landmark for the location of the carotid bifurcation.
Ibrahim et al., 2016 [20]	2 patients	Lateral neck dissection for cancer	1/2	73- and 58-year-old	Canada	1 male and 1 female	The existence of an anatomical variation in the position of the SAN in relation to the IJV through which the SAN passes in a fenestration in the IJV.
Laukontaus et al., 2017 [23]	37 patients	Chronic cerebrospinal venous insufficiency	4/5	23 to 59 years	Finland	28 females, 9 males	The US seems overly sensibles in terms of finding venous stenosis.
Taylor et al., 2016 [42]	11 patients	Techniques for evaluating hemodynamics through the IJV.	11/11	22 to 24 years	Japan	11 males	Hemodynamics can be evaluated by analyzing the cardiac variation in the IJV area in a minimally invasive manner.
Won et al., 2023 [35]	1 cadaver	Cephalic vein variation	100%	77-year-old	Korea	1 male	The possibility of using CV for invasive venous access is determined by the morpho-anatomical parameters; knowledge of its anatomical variations may be of considerable interest to doctors.
Mumtaz et al., 2019 [26]	1197 patients	Fenestration and duplication of the internal jugular vein (IJV) depending on the level at which it occurs.	40/1197	does not report	United Kingdom	does not report	Knowledge of variations in the anatomy of the internal jugular vein helps surgeons avoid complications during neck surgery and prevent morbidity.
Rusu et al., 2022 [32]	1 patient	Bilateral high fenestrations of the internal jugular vein.	100%	65-year-old	Romania	1 female	Such high IJV fenestrations require attention when central venous catheters are inserted. Elevated IJV fenestrations also require caution during surgical procedures targeting the parapharyngeal space or in high radical neck dissections.
Tokunaga et al., 2020 [33]	104 patients	Cardiac variation of the internal jugular vein	18% after donation	does not report	Japan	does not report	Cardiac variation in IJV should be considered a reliable indicator of intravascular volume loss and response to fluid administration.
Malcom et al., 2008 [24]	1 patient	Variation in the location of the IJV	100%	76 year old	United States	1 female	Doctors should be able to fully explore the anatomy of the neck using ultrasound to visualize the best location for cannulation. of the IJV in these patients.
Prades et al., 2002 [31]	3 patients	Duplication of the IJV.	100%	73, 52 and 62 years	France	3 males	The discovery of IJV duplication raises practical problems of surgical dissection during functional lymph node clearance while preserving neurovascular structures.
Yuan et al., 2017 [43]	210 patients	IJV and common carotid catheterization.	-	6 to 12 mean ages	China	83.33% this male	Conventional landmark-based internal jugular vein catheterization is difficult in pediatric patients.
Pegot et al., 2015 [30]	1 patient	Fenestration of the left internal jugular vein.	100%	58 years	France	1 female	The IJV is a common site for insertion of a central venous line, but in the case of fenestration, difficulties in catheter insertion could cause vascular injury along with cervical hemorrhage or hematoma.
Gardiner et al., 2002 [16]	1 patient	Anomalous relationship of the spinal accessory nerve to the internal jugular vein Anatomical variation between the common carotid artery and the internal jugular vein	100%	67 years	United Kingdom	1 male	During radical, modified radical, and selective neck dissections, level II nodes are almost always removed.
Umaña et al., 2015 [34]	78 patients	Anatomical variation between the common carotid artery and the internal jugular vein	15–54%	17–90 years	Colombia	44 males, 34 females	The internal jugular vein significantly overlapped the common carotid artery in 23 of 75 (30.7%).
Farina et al., 2013 [15]	Cases 313 patients with clinically MS Controls 298 Total 611 patients	Variation of the cross-sectional area of the internal jugular	veins with only VD (49.2%) and hypoplastic veins (7.5%). New morphological types of IJV, defined as “myopragic” (27.1%) and “myopragic with VD” (4.5%)	Cases 19 to 77 years controls 20–79 years	Italy	cases 137 men and 176 women Controls men/women 130/168	IJVs in MS patients detected hypoplastic veins (7.5%).
Burman et al., 2021 [10]	1 patient	Duplication of the internal jugular vein	100%	40 years	India	1 male	intraoperative discovery of a second medial vein and After the VJI it was identified with the same caliber and joining the IJV below the level of the omohyoid muscle
Abakay et al., 2019 [44]	1 patient	Fenestration of the internal jugular vein	100%	72 years	Turkey	1 male	In the operating room, a variation of a fenestration of the IJV with the AN was found incidentally was identified.
Deepak et al., 2015 [13]	3 patients	Variation in level of jugular vein bifurcation and position of internal jugular vein.	100%	35–50 years	India	3 females	Knowledge of these variations is important for surgeons to avoid accidental injuries and bleeding from the vein.
Lv et al., 2015 [22]	Internal jugular vein, inferior petrosal sinus (IPS), and their confluence pattern and implications for IPS catheterization.	Anatomical variations of the internal jugular vein (IJV), the inferior petrosal sinus (IPS), and their confluence pattern and implications for IPS catheterization.	-	-	China	-	It can be very useful for the neurointerventionist to avoid failure of transvenous embolization, avoiding procedures riskier and unnecessary.
Miccini et al., 2016 [25]	302 patients	Safety and Efficacy of Ultrasound-Guided CVP Placement Via the Right Internal Jugular Vein.	Four patients (1.3%) presented catheter malposition	Between 29 and 80 years old, with an average of 67.3.	Italy	176 males, 126 females.	No reported.
Nakayama et al., 2001 [28]	105 pediatric patients with congenital heart disease.	Venograms obtained from the right internal jugular vein (RIJV) to evaluate variations	Some disproportionately small vessels (defined as 3 mm for neonates and infants and %5 mm for older children) were seen in 8 patients (8%) and 3 patients had persistent left superior vena cava.	7 days to 10 years old.	Japan	does not report	The internal jugular vein may be useful for percutaneous cannulation.
Zivadinov et al., 2011 [37]	10 patients with MS	Role of venography in detection of abnormalities in the internal jugular vein	The sensitivity and specificity of Doppler ultrasound for detecting IJV abnormalities relative to catheter venography in patients with MS were calculated, respectively, at 82%, 100%.	35.4 ± 7.2 years	does not report	60% females and 40% males	Conventional MRV has limited value in evaluating IJV abnormalities for both diagnostic and posttreatment purposes.

IJV: internal Jugular vein; EJV: External jugular vein; SAN: Spinal accessory nerve; US: Doppler ultrasound; CV: Cephalic vein; CFV: Common facial vein; VD: Valvular defect. CVST: Cerebral venous sinus thrombosis; IPS: inferior petrosal sinus; CVP: central venous port; RIJV: Right internal jugular vein; MS: Multiple sclerosis; MRV: Venography FV: facial vein; CCA: Common carotid artery.

**Table 2:** Articles included in the prevalence meta-analysis [1,14,15,17,18,22,25,26,28,38].

Author	Total n	Prevalence
Asouhidou, 2008, [38]	93	3
Denys, 1991, [14]	200	17
Wang, 2020, [1]	221	2
Hameedullah, 2001, [18]	200	14
Gupta, 2003, [17]	89	8
Lv, 2015, [22]	1000	4
Mumtaz, 2019, [26]	1197	40
Farina, 2013, [15]	313	25
Miccini, 2016, [25]	302	4
Nakayama, 2001, [28]	105	8

**Table 3:** Subgroups analysis [1,14,15,17,18,22,25,26,28,38].

Parameters	Number of Studies and Subjects	Prevalence	95% CI	I2	*p* Value
Overall	10 (3720)	3.36	2.81–6.96	94.46%	-
Cadaveric	3 (1182)	3.12	1.87–8.16	83.62%	*p* = 0.165
Imaging	7 (2538)	5.33	2.12–7.31	91.58%	
Asia	5 (1615)	4.44	1.83–8.22	94.42%	*p* = 0.873
Africa	0 (0)	-	-	-	
Europe	4 (1905)	4.88	1.87–6.12	88.73%	
America	1 (200)	8.5	-	-	
Oceania	0 (0)	-	-	-	
Left side	3 (403)	4.44	1.11–8.92	78.62%	*p* = 0.212
Right side	4 (696)	6.18	2.11–9.45	77.84%	
Male	5 (672	2.52	1.82–4.11	79.93%	*p* = 0.321
Female	6 (432)	1.38	0.65–2.51	91.22%	
Unilateral	2 (1089)	1.65	0.99–2.35	66.11%	*p* = 0.455
Bilateral	1 (89)	1.12	-	-	

## Supplementary Materials

The following supporting information can be downloaded at: https://www.mdpi.com/article/10.3390/diagnostics14232765/s1, Table S1: Searches for strategies.
