# Supplementary material for: Systematic Review and Meta-Analysis of Internal Jugular Vein Variants and Their Relationship to Clinical Implications in the Head and Neck
Source: Diagnostics (Basel). 2024 Dec 9;14(23):2765. doi: 10.3390/diagnostics14232765 (PMC11640428; doi:10.3390/diagnostics14232765)
Supplement: Supplementary file 1 [file diagnostics-14-02765-s001.zip › diagnostics-3262610-supplementary.pdf]

Supplementary Table S1: Details of the search strategy

Database	Search strategy	Results	
		**20-07-24**	**13-11-2024**
Medline	**neuropathic pain[Title] AND Cancer[Title]** Filters: **Humans** ("neuropathic pain"[Title] AND "Cancer"[Title]) AND (humans[Filter])	59	61
Scopus	**neuropathic pain[Title] AND Cancer[Title]** Filters: **Humans** ("neuropathic pain"[Title] AND "Cancer"[Title]) AND (humans[Filter])	22	22
WOS	variants gastric vein OR aberrant gastric vein AND clinical anatomy OR clinical treatment	13	13
Google scholar	variants gastric vein OR aberrant gastric vein AND clinical anatomy OR clinical treatment	44	44
Cinahl	variants gastric vein OR aberrant gastric vein AND clinical anatomy OR clinical treatment	7	7
Lilacs	variants gastric vein OR aberrant gastric vein AND clinical anatomy OR clinical treatment	4	4
	Total	149	151

* All searches were carried out on November 13, 2024.
